# Ultrasonographic measurement of the collateral ligaments of the distal interphalangeal joint in the Argentine Polo Horse

**DOI:** 10.29374/2527-2179.bjvm003125

**Published:** 2025-11-13

**Authors:** Geórgia Camargo Góss, Fabricio Desconsi Mozzaquatro, Ingrid Rios Lima Machado, Etiele Maldonado Gomes, Natálie Rodrigues Martins, Claudia Acosta Duarte

**Affiliations:** 1 Programa de Pós-Graduação em Ciência Animal, Departamento de Clínica e Cirurgia de Grandes Animais (DCCGA), Hospital Universitário Veterinário (HUVet), Universidade Federal do Pampa (UNIPAMPA), Campus Uruguaiana, RS, Brazil.; 2 Departamento de Ciências Agrárias e Ambientais, Faculdade de Medicina Veterinária, Universidade Estadual de Santa Cruz, Ilhéus, Brazil.; 3 Departamento de Reprodução Equina (DRE), HUVet, UNIPAMPA, Campus Uruguaiana, RS, Brazil.; 4 Departamento de Diagnóstico por Imagem (DDI), HUVet, UNIPAMPA, Campus Uruguaiana, RS, Brazil.; 5 Programa de Pós-Graduação em Ciência Animal, Departamento de Clínica e Cirurgia de Pequenos Animais (DCCPA), HUVet, UNIPAMPA, Campus Uruguaiana, RS, Brazil.; 6 Programa de Pós-Graduação em Ciência Animal, DCCGA, HUVet, UNIPAMPA, Campus Uruguaiana, RS, Brazil.; 7 DCCGA, HUVet, UNIPAMPA, Campus Uruguaiana, RS, Brazil.

**Keywords:** cross-sectional area, measurement, asymmetry, ultrasound, área da secção transversal, mensuração, assimetria, ultrassom

## Abstract

The collateral ligaments of the distal interphalangeal joint (CL-DIPJ) are important structures for athletic horses. Ligament injuries can cause lameness, but ultrasound imaging can detect such lesions, which usually present with changes in echogenicity and an increase in the cross-sectional area (CSA) of the ligament. Measurements of these structures vary among authors, which complicates the diagnosis. Thus, this study aimed to describe the ultrasound measurements (dorsopalmar diameter [DPD], lateromedial diameter [LMD], and CSA) of the CL-DIPJ in Argentine Polo horses. We measured the CL-DIPJ of 25 Argentine Polo horses. The measurements were repeated three times, and the mean value for the selected variables was calculated. The Student's t-test was applied to compare the contralateral ligaments and, subsequently, the lateral (LCL) and medial (MCL) collateral ligaments, regardless of the thoracic limb assessed. There was no difference (p> 0.05) between the contralateral ligaments. Similarly, no difference was found between the LCL and MCL for DPD and LMD. Regarding the CSA, there was a difference (p < 0.05). Thus, the mean values for the CL-DIPJ of the thoracic limbs of Argentine Polo horses were 13.13 ± 0.83 mm for the DPD, 8.79 ± 0.57 mm for the LMD, and a cross-sectional area of 0.97 ± 0.06 cm^2^ for the LCL and 0.98 ± 0.05 cm^2^ for the MCL.

## Resumo

Os ligamentos colaterais da articulação interfalangeana distal (CL-DIPJ) são estruturas importantes para cavalos atletas. Lesões ligamentares podem causar claudicação, mas a ultrassonografia é capaz de detectar tais lesões, onde geralmente são observadas alterações na ecogenicidade e aumento da área de secção transversa do ligamento (CSA). As medidas dessas estruturas diferem entre os autores, prejudicando o diagnóstico. Assim, o objetivo deste estudo foi descrever as medidas ultrassonográficas do diâmetro dorsopalmar (DPD), diâmetro lateromedial (LMD) e CSA da CL-DIPJ de cavalos de polo argentino. Para isso, foram utilizados 25 cavalos de polo argentino, os quais foram submetidos à medição da CL-DIPJ. As medições foram repetidas três vezes e, após, foi calculado o valor médio para as variáveis ​​selecionadas. O teste t de Student foi aplicado para comparar os ligamentos contralaterais e, posteriormente, os ligamentos colaterais laterais (LCL) e mediais (MCL) independentemente do membro torácico. Não houve diferença (p> 0,05) entre os ligamentos contralaterais. O mesmo foi encontrado entre ligamentos colaterais laterais e mediais, considerando os diâmetros dorsopalmar e lateromedial. Em relação ao CSA, houve diferença (p<0,05). Assim, os valores médios para o CL-DIPJ dos membros torácicos dos cavalos de polo argentino foram de 13,13 ± 0,83 mm para o DPD, 8,79 ± 0,57 mm para o LMD e uma área de secção transversa de 0,97 ± 0,06 cm2 para LCL e 0,98 ± 0,05 cm2 para MCL.

## Introduction

Polo is a sport practiced worldwide. In Brazil, the south and southeast regions have the greatest number of practitioners. The most common breeds include the Criollo, Thoroughbred, American Quarter Horse, and the Arabian, as well as their crosses. The Argentine polo horse emerged from the crossing of the Criollo and Thoroughbred breeds. This resulted in a rustic, fast horse with a great capacity for learning and skill in executing the movements necessary for the sport. Furthermore, they inherited morphological characteristics from both breeds, with an average size and weight between that of the Criollo and the Thoroughbred.

The practice of this sport requires sudden stops and high-speed turns, predisposing the animals to injuries (Pfau et al., 2016). Among them is desmitis of the collateral ligaments of the distal interphalangeal joint. These ligaments are small structures that contribute to joint stability. When injured, horses present with acute lameness of varying degrees and show different responses to flexion tests and perineural blocks (Turner & Sage, 2002). This condition is mainly caused by the exacerbation of collateral movements of the middle and distal phalanges, which occurs during irregular hoof support (Denoix, 1998).

The diagnosis of CL-DIPJ desmitis is based on the patient's history and clinical signs, in addition to complementary methods (Trope & Whitton, 2009). Imaging modalities such as magnetic resonance and ultrasonography have been used for diagnosis (Dyson et al., 2004). In the ultrasound exam, the main changes found are related to the increase in the cross-sectional area of the ligament (CSA) and changes in echogenicity (Denoix et al., 2011a). However, ultrasound measurements of the CL-DIPJ differ among authors and breeds (Contreras, 2009; Denoix et al., 2011b; Góss et al., 2018; Ribeiro, 2016; Turner & Sage, 2002), which can lead to diagnostic errors when using CSA for diagnosis.

Therefore, the aim of this study was to describe the ultrasound measurements of the collateral ligaments of the distal interphalangeal joint of Argentine Polo horses.

## Materials and methods

This study was approved by the Ethics Committee on the Use of Animals of UNIPAMPA under protocol 027/2017. Twenty-five Argentine Polo horses were used (18 mares and seven geldings), with an average age of 8 ± 3 years. The average weight and height were 470 ± 28 kg and 153 ± 4.47 cm, respectively. All animals underwent general and specific physical assessment of the locomotor system. The general physical exam consisted of assessing mucosal color and temperature, measuring capillary refill time, and monitoring heart and respiratory rates. For the evaluation of the locomotor system, static and dynamic inspection were performed. No abnormalities were observed in either evaluation. In addition, as informed by the veterinarian responsible for the animals, none of the horses had a history of lameness in the hoof region.

**Figure 1:**
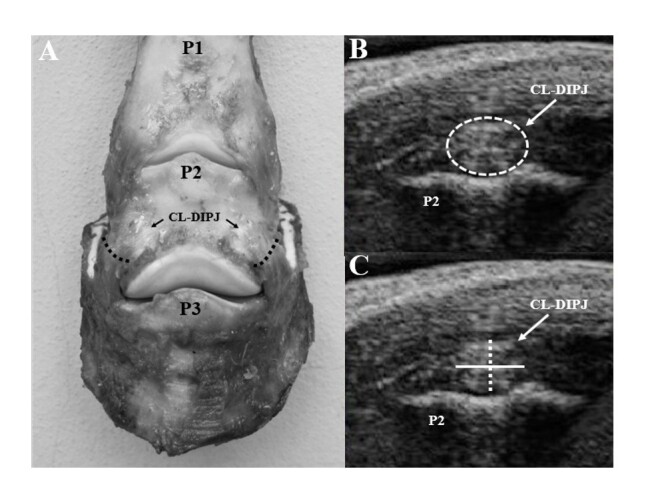
(A) Anatomical image of the equine digit showing the proximal (P1), middle (P2) and distal (P3) phalanges. The collateral ligaments of the distal interphalangeal joint (CL-DIPJ) are highlighted by the black arrows. The dotted black lines demonstrate the transducer positioning regions for imaging of the medial and lateral CL-DIPJ; (B) Ultrasound image demonstrating the measurement of CSA of the CL-DIPJ (white dotted line); (C) Ultrasound image demonstrating the measurement of DPD (white line) and LMD (white dotted line) of the CL-DIPJ.

For ultrasound examination, the horses were restrained by the halter. Trichotomy was performed on the dorsolateral and dorsomedial regions of both thoracic limbs, slightly dorsal to the coronary band. The ultrasound device (Chison 8300VET) used was equipped with a linear transducer adjusted to 6 MHz. The transducer was positioned (Figure 1A) as previously described by Denoix et al. (2011b). For the measurement, transverse images were obtained using the coronary band as an acoustic window, considering the anatomical location of the CL-DIPJ (Figure 1B).

The ligament was measured only when the CL-DIPJ appeared in the ultrasound image as echogenic, oval structures located in the concavity of the distal edge of the middle phalanx. Subsequently, three images of each ligament were obtained in this position. In each of them, the dorsopalmar diameter (DPD), lateromedial diameter (LMD), and cross-sectional area (CSA) were measured (Figures 1B and 1C). Afterwards, the mean values were calculated for each of the variables.

For statistical analysis, the SPSS Statistic 20^®^ statistical software was used. The significance level was set at p < 0.05, and the Student's t-test was applied. All values shown are expressed as mean ± standard deviation. First, the Student's t test was applied to compare the contralateral ligaments and then the lateral and medial ligaments, regardless of the thoracic limb assessed.

## Results

Table 1 describes the mean values obtained for the ultrasound measurements of the medial and lateral CL-DIPJ of the thoracic limbs. No statistical difference (p > 0.05) was observed between contralateral ligaments (left vs. right) for any of the three variables.

**Table 1:** Mean ultrasonographic measurements of the lateral and medial collateral ligaments of the distal interphalangeal joint in the thoracic limbs of Argentine polo horses.

	**Left thoracic limb**		**Right thoracic limb**	
	**Lateral**	**Medial**	**Lateral**	**Medial**
**Dorsopalmar diameter**	13.19 ± 0.65 mm	12.99 ± 0.71 mm	13.11 ± 0.74 mm	13.31 ± 1.03 mm
**Lateromedial diameter**	8.79 ± 0.72 mm	8.84 ± 0.71 mm	8.64 ± 0.46 mm	8.89 ± 0.74 mm
**Cross-sectional area**	0.97 ± 0.06 cm^2^	0.98 ± 0.05 cm^2^	0.97 ± 0.05 cm^2^	0.98 ± 0.04 cm^2^

* p>0.05. [[Q1: Q1]]

Considering the similarity between the contralateral collateral ligaments, the mean values of the lateral and medial ligaments were compared, regardless of the thoracic limb assessed. In this evaluation, there was a significant difference in the cross-sectional area (p < 0.05), but the other parameters (dorsopalmar and lateromedial diameter) did not differ significantly (p > 0.05). Thus, a mean value of 13.13 ± 0.83 mm was obtained for the dorsopalmar diameter and 8.79 ± 0.57 mm for the lateromedial diameter. For the CSA, the mean value was 0.97 ± 0.06 cm^2^ for the lateral collateral ligaments and 0.98 ± 0.05 cm^2^ for the medial collateral ligaments.

## Discussion

When comparing Argentine polo horses with Criollo horses, it was expected that their ligament sizes would be similar, since the former originated from crossing Criollos with Thoroughbreds. However, the mean values obtained in adult Criollo horses with an average weight of 392 kg and height of 1.42 m were 9.67 mm for dorsopalmar diameter, 6.94 mm for lateromedial diameter, and 0.52 cm^2^ for cross-sectional area (Góss et al., 2018). Thus, the CL-DIPJ of Argentine polo horses were larger than those of the Criollos in all variables measured in this study. In Purebred Chilean horses—which have a similar origin, size, and conformation to Criollos—the dorsopalmar diameter values (12.7-13 mm) were similar to those of the Argentine Polo horses. The cross-sectional area (0.55-0.61 cm^2^) and LMD (5.4-5.9 mm) were lower in the Purebred Chilean horses (Contreras, 2009). Considering that the horses in this study had a higher average weight and height than the Criollo horses (Góss et al., 2018), this suggests that smaller horses have smaller ligaments. Thus, care should be taken when assessing the CL-DIPJ in smaller horse breeds to avoid diagnostic errors related to an increased CSA.

In American Quarter horses with an average weight of 450 kg (Ribeiro, 2016), the ultrasound imaging measurements described were 11.7 ± 1.4 mm for the dorsopalmar diameter, 7 ± 0.9 mm for the lateromedial diameter and 0.77 ± 0.17 cm^2^ for CSA. Thus, the Argentine Polo horses also showed larger measurements than the Quarter Horses with the average weight described. Similarly, the average CSA of the Paint Horse, Quarter Horse, and Arabian breeds (0.61-0.65 cm^2^) reported by Turner and Sage (2002) was lower than that of the Argentine Polo horses. Furthermore, in a study with horses weighing 550 kg, Denoix et al. (2011b) described that DPD values can vary between 12-16 mm, LMD between 6-9 mm, and CSA between 0.6-0.9 cm^2^. The values found in the present study are within the reference range for dorsopalmar and lateromedial diameters. The cross-sectional area in the Argentine Polo breed was slightly higher than this reference range.

The larger size of the medial CL-DIPJ is a recurrent observation in other studies involving this parameter (Góss et al., 2018; Ribeiro, 2016); however, no statistical difference was detected between them. This finding reinforces that a comparative evaluation should be made with the contralateral ligament, not between ligaments of the same limb, to ensure a more reliable diagnosis.

The ultrasound measurement values of the horses studied were larger than those previously observed both in heavier horses (Denoix et al., 2011b) and in lighter ones (Góss et al., 2018; Ribeiro, 2016). Studies involving ultrasonographic anatomy have great clinical relevance, especially for soft tissues. This is because inflammatory, degenerative, and/or traumatic changes increase the CSA of the affected structures, primarily due to the accumulation of inflammatory fluid. Therefore, standardizing these measurements across breeds of different sizes can help veterinary clinicians identify and monitor CL-DIPJ desmitis in their routine practice.

Thus, this suggests that weight may not be a reliable morphometric parameter for determining the size of the CL-DIPJ. Additionally, it is suggested that withers height can be considered for this purpose. Unfortunately, the studies cited do not provide this information.

## Conclusions

This study established the mean values for the collateral ligaments of the distal interphalangeal joint in Argentine Polo horses. Ligament measurements for this breed showed that the cross-sectional area of the medial collateral ligament was greater than that of the lateral collateral ligament.

The mean values for the collateral ligaments of the distal interphalangeal joint in the thoracic limbs of Argentine Polo horses were: dorsopalmar diameter, 13.13 ± 0.83 mm; lateromedial diameter, 8.79 ± 0.57 mm; and cross-sectional area, 0.97 ± 0.06 cm^2^ for the lateral and 0.98 ± 0.05 cm^2^ for the medial ligaments.

## References

[B001] Contreras R. A. C. (2009). Caracterización ecotomográfica de los ligamentos colaterales de la articulación interfalángica distal en el equino Pura Sangre Chileno..

[B002] Denoix J. M. (1998). Ultrasonographic examination of the distal interphalangeal joint collateral ligaments in horses. Ippologia.

[B003] Denoix J. M., Dupays A.-G., Bertoni L., Werpy N., Audigié F. (2011). Ultrasonographic examination of the collateral ligaments of distal interphalangeal joint in horses. Part B: Abnormal findings and lesions. Equine Veterinary Education.

[B004] Denoix J. M., Bertoni L., Heitzmann A. G., Werpy N., Audigié F. (2011). Ultrasonographic examination of the collateral ligaments of distal interphalangeal joint in horses. Part A: Technique and normal images. Equine Veterinary Education.

[B005] Dyson S. J., Murray R., Schramme M., Branch M. (2004). Collateral desmitis of the distal interphalangeal joint in 18 horses (2001-2002). Equine Veterinary Journal.

[B006] Góss G. C., Duarte C. A., Mozzaquatro F. D., Döwich G., Klaus G., Altermann O., Severo E. S., Nunes O. G., Bataglin C. F. (2018). Ultrasonographic measurement of collateral ligaments of the distal interphalangeal joint in Criollo horses. Semina: Ciências Agrárias.

[B007] Pfau T., Parkes R. S., Burden E. R., Bell N., Fairhurst H., Witte T. H. (2016). Movement asymmetry in working polo horses. Equine Veterinary Journal.

[B008] Ribeiro G. H. C. (2016). Avaliação ultrassonográfica dos ligamentos colaterais da articulação interfalangeana distal do membro torácico em equinos Quarto de Milha..

[B009] Trope G. D., Whitton R. C. (2009). Medial collateral ligament desmitis of the distal interphalangeal joint in the hindlimb of a horse: Treatment with cast immobilization. Australian Veterinary Journal.

[B010] Turner T. A., Sage A. M. (2002). Desmitis of distal interphalangeal collateral ligaments: 22 cases..

